# Fibromodulin – A New Target of Osteoarthritis Management?

**DOI:** 10.3389/fphar.2019.01475

**Published:** 2019-12-10

**Authors:** Chenshuang Li, Pin Ha, Wenlu Jiang, Christos S. Haveles, Zhong Zheng, Min Zou

**Affiliations:** ^1^Key Laboratory of Shaanxi Province for Craniofacial Precision Medicine Research, College of Stomatology, Xi’an Jiaotong University, Xi’an, China; ^2^Department of Orthodontics, College of Stomatology, Xi’an Jiaotong University, Xi’an, China; ^3^Division of Growth and Development, Section of Orthodontics, School of Dentistry, University of California, Los Angeles, Los Angeles, CA, United States; ^4^Department of Orthodontics, School of Dental Medicine, University of Pennsylvania, Philadelphia, PA, United States; ^5^David Geffen School of Medicine at University of California, Los Angeles, Los Angeles, CA, United States

**Keywords:** fibromodulin, arthritis, osteoarthritis, collagen, inflammation, keratan sulfate

## Introduction

As a leading cause of disability among adults, osteoarthritis (OA) leads to serious public health and economic burdens. Currently, treatment options for OA are generally based on symptom severity and duration, with the goals of symptom alleviation and improvement in functional status (Taruc-Uy and Lynch, 2013). Nonpharmacologic and pharmacologic strategies are used initially, while a surgical approach to OA is reserved for chronic cases when these treatments failed. Unfortunately, the currently available clinical pharmacologic treatments for OA, such as analgesia, glucocorticoids, non-steroidal anti-inflammatory drugs, and disease-modifying antirheumatic drugs, are not adequately effective (Chevalier et al., 2009; Scott, 2010; Verbruggen et al., 2012; Chevalier et al., 2015; Appleton, 2018; Li and Zheng, 2018; Li et al., 2018), and generally associated with a diversity of adverse side-effects (Habib et al., 2010; Cooper et al., 2016; Compston, 2018). For instance, analgesia does not reduce inflammation and cartilage damage (Appleton, 2018), glucocorticoids have been reported to induce severe damages in the musculoskeletal, cardiovascular, and gastrointestinal systems (Cooper et al., 2016; Compston, 2018), and non-steroidal anti-inflammatory drugs do not actively control arthritis progression (Appleton, 2018). Accumulating evidence demonstrates that an ideal OA-combating agent should be able to reduce inflammation and promote cartilage regeneration safely, which has long been desired. In responding to this demand, the current strategy for disease-modifying osteoarthritis drug seeking has shifted to biologoical molecules that promote chondorgenic development and regenration.

To date, a diversity of well-known pro-chondrogenic growth factors, such as bone morphogenetic proteins (BMPs) and transforming growth factors (TGFs), have been examined for OA treatment. However, the results are not optimistic since intra-articular injection of these growth factors could even enhance the inflammatory infiltration in damaged joints (Allen et al., 1990; Fava et al., 1991; Hong et al., 2009). Meanwhile, multiple transcriptional factors that potentially suppress inflammation, such as nuclear factor of activated T cells 1 (NFATc1), NFATc2, and runt-related transcription factor 1 (RUNX1), have also be introduced in this arena against OA, while they do not hold much promise presently. For example, the function of NFATc proteins in arthritis is controversial (Yaykasli et al., 2009; Miclea et al., 2011; Greenblatt et al., 2013).

Another possibility for fighting OA is utilizing the extracellular matrix (ECM) molecules that naturally distribute in the articular cartilage. For example, fibromodulin (FMOD) is an ECM protein with multiple keratin sulfate side-chains that belongs to the small leucine-rich proteoglycan family (Plaas et al., 1990). It was first identified as a collagen-binding molecule broadly distributed in connective tissues, with particularly high expression in cartilage (Hedbom and Heinegard, 1989). In the past three decades, in addition to the broad attention of its effects on collagen fibrillogenesis (Chen et al., 2010), muscle development (Lee et al., 2018a; Lee et al., 2018b), cell reprogramming (Zheng et al., 2012; Li et al., 2016; Zheng et al., 2019), angiogenesis (Jian et al., 2013; Zheng et al., 2014; Ao et al., 2017), wound healing (Zheng et al., 2017), and tumorigenesis (Pourhanifeh et al., 2019), the involvement of FMOD in cartilage development and maintenance as well as arthritis progression, especially in temporomandibular joint (TMJ) OA, has been investigated through world-wide collaboration. Here, we review the current research investigating FMOD and arthritis, and aim to provide novel insight into the potential use of FMOD for OA management.

## Spatiotemporal Distribution of FMOD During Cartilage Growth and Development

An investigation that focused on mouse glenohumeral joints demonstrated that, at 12–13 days post-coitus when the limb buds are just condensing mesenchymal cells, FMOD was not detectable at the protein level (Murphy et al., 1999). Intense FMOD staining was first noticed at the surface of the scapular and humeral anlage intracellularly and pericellularly in the interzone at 14–15 days post-coitus (Murphy et al., 1999). Starting from 17 days post-coitus, a strong FMOD signal was found in the ECM surrounding the chondrocytes at the surface of the joints and proliferating chondrocytes in the epiphyses of the humerus and scapula (Murphy et al., 1999). Meanwhile, during postnatal maturation until adulthood, FMOD was detected throughout the ECM of the developing articular surface and the growth plate but was more abundant in articular cartilage (Murphy et al., 1999). Since FMOD was associated with prechondrocytic mesenchymal cells in the interzone before joint cavitation and with developing articular chondrocytes in the maturing and young adult limbs, it has been proposed that FMOD may function in the early genesis of articular cartilage (Murphy et al., 1999).

It is worth noting that FMOD shared a similar temporospatial transcriptional pattern with type II collagen in mouse knee joints during postnatal development, while *FMOD* gene expression reached the maximum level at 1 month old (Saamanen et al., 2001). *FMOD* transcription was restricted to chondrocytes and peaked in the proliferating zone and the early articular cartilage (Saamanen et al., 2001), which had been confirmed at the protein level by immunostaining (Murphy et al., 1999). In mature animals, *in situ* hybridization revealed that both pericellular and interterritorial cartilage at knee joints had high *FMOD* expression with the highest intensity in the middle and deep zones of the uncalcified cartilage (Saamanen et al., 2001). At 6 months old, FMOD staining decreased in the uncalcified cartilage but increased in the calcified cartilage (Saamanen et al., 2001). FMOD was also detected in the hypertrophic chondrocytes of the secondary ossification centers and growth plate of mice at 10 days old, and transcription of *FMOD* was diminished and finally disappeared with maturation and aging of the trabecular epiphyses (Saamanen et al., 2001).

## Structural Alteration of FMOD in Aging and Arthritis Progression

In addition to its spatiotemporal distribution, FMOD’s structural heterogeneity was also noticed during articular cartilage growth and development. For instance, FMOD isolated from young articular cartilage carries neither α(2-6)-linked *N*-acetylneuraminic acid nor α(1-3)-linked fucose in the N-linked keratan sulfate chains (Lauder et al., 1996). Meanwhile, an age-related increase has been observed in the abundance of both α(2-6)-linked *N*-acetylneuraminic acid and α(1-3)-linked fucose, but not the levels of galactose sulfation (Lauder et al., 1998). Western blot showed FMOD-derived from fetal and neonatal articular cartilage (f/n-FMOD) as a diffused region with a relative molecular weight of 70–110 kDa (Cs-Szabo et al., 1995; Roughley et al., 1996), while FMOD-derived from mature adult (a-FMOD) was a more discrete component with a relative molecular weight of 67 kDa (Cs-Szabo et al., 1995; Roughley et al., 1996)—larger than the FMOD core protein without post-translational modifications (46 kDa). Interestingly, digesting f/n-FMOD with keratanase II or endo β-galactosidase reduces its molecular weight to a similar level of a-FMOD (Cs-Szabo et al., 1995). Thus, Roughley et al. argued that FMOD might predominantly exist in the proteoglycan form in juvenile cartilage tissues but is mainly in a glycoprotein form in the adult counterparts (Roughley et al., 1996).

Interestingly, FMOD is one of the small leucine-rich proteoglycans with the most significantly increased protein fragmentation in arthritis compared with macroscopically healthy articular cartilage from the age-matched donors (Melrose et al., 2008). In addition to the 59 kDa band, multiple small bands can be detected by Western blot when FMOD is isolated from articular cartilage of OA and rheumatoid arthritis patients (Cs-Szabo et al., 1995; Roughley et al., 1996; Melrose et al., 2008; Shu et al., 2019). Moreover, when using N-glycosidase to remove the sulfate chains from FMOD isolated from arthritic articular cartilage, several protein bands with the size of 43, 40, and 27 kDa were detected (Cs-Szabo et al., 1995). Therefore, arthritis progression may not only alter the degree and type of its carbohydrate substation but also lead to the breakage of the FMOD core protein.

Meanwhile, degradation of FMOD core protein was also observed in interleukin (IL)-1-challenged cartilage (Sztrolovics et al., 1999; Shu et al., 2019)—a representative model that elucidates the genetic and molecular pathogenesis of inflammation-related secondary OA (Kuyinu et al., 2016). The degradation of FMOD core protein was predominantly catalyzed by matrix metalloproteinases (MMPs) and ADAM metallopeptidases with thrombospondin type 1 motifs (ADAMTSs) (Kashiwagi et al., 2004; Shu et al., 2019). *In vitro* digestion of healthy human knee cartilage with MMP-13, ADAMTS-4, and ADAMTS-5 generated FMOD fragments of similar sizes as FMOD derived from OA cartilage without digestion (Shu et al., 2019). Notably, the fragmented FMOD is always detected by the antibody recognizing the N-terminal fragment of FMOD but not the one recognizing the C-terminal (Melrose et al., 2008; Shu et al., 2019). One possible explanation is that the C-terminus is vulnerable to the fragmentation and not stably retained in the tissue, and substantially lost into the synovial fluid (Melrose et al., 2008). Importantly, MMP-13 degradation of FMOD resulted in a fragment of 30 kDa, which was also detected in moderately and severely fibrillated cartilage, instead of healthy or slightly fibrillated cartilage (Monfort et al., 2006). These phenomena may support the hypothesis that the sensitivity of FMOD protein fragmentation is increased along with the severity of cartilage degradation.

## Lessons From FMOD Deficient Mice for OA Investigation

FMOD-null (*Fmod* ^−^ *^/^* ^−^) mice have distinct knee joints in comparison with their wildtype (WT) littermates at 36 weeks old (Gill et al., 2002), accompanied by a significantly higher histological arthritis score (Ameye et al., 2002). In addition, serial sections through FMOD-null mice knees showed degeneration and joint remodeling histologically. More severe incidences of degeneration occurred in the area of the tibial condyles that are uncovered by the menisci, as these sites experience the highest loading stress, resulting in considerable loss of cartilage and bone thickness (Gill et al., 2002). Moreover, the menisci of FMOD-null mice had a markedly less sharp profile with more rounded edges, similar to FMOD-null ligaments, which were also more likely to be damaged compared to WT ligaments. The area of tibial articular cartilage was even more exposed due to degenerated menisci compared to that of the WT littermates (Gill et al., 2002). Furthermore, knee joints of *Fmod* ^−^ *^/^* ^−^ mice at 80 weeks old displayed full-depth lesions of articular cartilage and clusters of cells that were not seen in the knee joints of WT littermates (Gill et al., 2002).

As biglycan (BGN) and FMOD have overlapping and possible compensatory functions in the joints (Shirakura et al., 2017), *BGN* and *FMOD* double-knockout (*Bgn* *^−/0^* */Fmod* *^−/−^*) mice exhibit an earlier onset of OA than *Fmod* ^−^ *^/^* ^−^ mice. *Bgn* ^−^ *^/0^* */Fmod* ^−^ *^/^* ^−^ mice presented with an abnormal gait characterized by the decreased flexibility of knee and ankle joints (dragging leg), which was observed as early as 3 weeks old. Additionally, at 3 months old, the histological arthritis score of the *Bgn* ^−^ *^/0^* */Fmod* ^−^ *^/^* ^−^ knee joints was significantly higher than that of the WT knee joints. However, the abnormal gait phenomena were observed in neither *BGN* nor *FMOD* single knockout mice (Ameye et al., 2002).

Moreover, BGN and FMOD are also highly expressed in the disc and articular cartilage of the TMJ (Wadhwa et al., 2005a). *Bgn* ^−^ *^/0^* */Fmod* ^−^ *^/^* ^−^ mice developed accelerated OA accompanied by small vertical clefts in the condylar cartilage and partial disruption of the disc as compared to WT animals at 6 months old (Wadhwa et al., 2005b). At 18 months old, extensive cartilage erosion was visible in the *Bgn* ^−^ *^/0^* */Fmod* ^−^ *^/^* ^−^ mice TMJ (Wadhwa et al., 2005b).

## Potential Roles of FMOD in Arthritis

There are several hypotheses about the possible roles of FMOD in arthritis. FMOD binds to collagens (Melching and Roughley, 1999), and fragmentation of FMOD during arthritis progression may destabilize collagen fibrils, rendering them more susceptible to tissue collagenases (Kashiwagi et al., 2004). However, such a difference between WT and FMOD-null mice may not necessarily have immediately visible effects at the ultrastructural level in adults (Ameye et al., 2002).

Alternatively, FMOD may sequester TGF-β/BMP superfamily members in the ECM and thereby prevent their binding to the cellular receptors (Wadhwa et al., 2005a). For example, when treating the TMJ with BMP2, both catabolic and anabolic markers were more profoundly upregulated in the *Bgn* ^−^ *^/0^* */Fmod* ^−^ *^/^* ^−^ mice than WT animals (Shirakura et al., 2017). This observation suggests that BGN and FMOD could protect the condyle from BMP2-induced matric turnover (Shirakura et al., 2017). Additionally, the sequestration of TGF-β1 in mandibular condylar chondrocyte ECM decreased in *Bgn* ^−^ *^/0^* */Fmod* ^−^ *^/^* ^−^ mice. The overactive TGF-β1 signal transduction in *Bgn* ^−^ *^/0^* */Fmod* ^−^ *^/^* ^−^ mice accelerated both production and degradation of type II collagen and aggrecan, and subsequently led to an overall imbalance in ECM turnover that favors cartilage degradation and the onset of OA (Embree et al., 2010).

FMOD may also function as a barrier preventing cell adhesion and subsequent cartilage damage. For example, FMOD administration dramatically prevents the adhesion of polymorphonuclear neutrophils and fibroblasts on articular cartilage surfaces (Noyori and Jasin, 1994; Mitani et al., 2001). This inhibition of cellular attachment may be attributed to the capability of FMOD to mask epitopes of cartilage collagen that face the joint cavity (Noyori and Jasin, 1994).

Furthermore, FMOD may participate in arthritis progression by directly manipulating inflammatory reactions. For instance, C1q and complement inhibitor factor H can directly bind to FMOD but in different regions (Akimoto et al., 2006). However, the deposition of the membrane attack complex and C5a release were lower in the presence of FMOD, presumably due to the formation of the FMOD-factor H complex (Akimoto et al., 2006). Interestingly, IL-1 only stimulates the binding of C1q, but not factor H, to the N-terminal fragment of FMOD in cartilage (Akimoto et al., 2006). Thus, FMOD may balance the activation of the classical complement pathway: when maintained in its intact form, FMOD silences the complement cascade by binding factor H; on the other hand, when FMOD is degraded or fragmented, as seen in OA (Melrose et al., 2008; Shu et al., 2019), the N-terminal FMOD segment binds to C1q and in turn activates the complement system to eliminate pathogens and damaged cells for tissue recovery and reconstruction.

FMOD has been used as an early marker of chondrogenesis (Barry et al., 2001). The expression level of FMOD is inversely correlated with the passage number of human chondrocytes in monolayer cultivation (Lin et al., 2008). In the TMJ cartilage of 3-month-old *Bgn* ^−^ *^/0^* */Fmod* ^−^ *^/^* ^−^ mice, fewer proliferative chondrocytes were noticed in comparison to that of their WT counterparts (Wadhwa et al., 2005b). Moreover, *Bgn* ^−^ *^/0^* */Fmod* ^−^ *^/^* ^−^ mice presented with more chondrocyte apoptosis in the articular cartilage than WT mice at the same developmental stage (Wadhwa et al., 2005a). A recent study even showed that microRNA-340-5p negatively regulated OA chondrocyte proliferation while stimulating apoptosis by reducing *FMOD* expression (Zhang et al., 2018). Nevertheless, the exact function of FMOD in chondrogenesis has yet to be fully uncovered.

## Further Direction

As aforementioned, FMOD is a critical ECM component involved in articular cartilage development, growth, aging, and arthritis; however, the exact functions of FMOD during arthritis are still unclear. Take advantage of the development of the Cre/Lox as well as CRISPR-Cas9 recombination system, the specific functions of FMOD during arthritis progression could be deciphered in detail with tissue-specific knockout animal models. Recently, it has been reported that FMOD can be successfully produced and purified from the cell culture supernatant of stable recombinant CHO-K1 cells transfected with a plasmid harboring the human *FMOD* gene (Zheng et al., 2012; Li et al., 2016; Pourhanifeh et al., 2019). Since FMOD whole protein is now easy to produce, further in-depth investigations are warranted to reveal the underlying mechanism of action of FMOD as a new generation disease-modifying osteoarthritis drug candidate. Last but not least, the plasmid- or virus-mediated expression, as well as directly synthesis, could be utilized to identify the functional sequence(s) of FMOD that regulate(s) cartilage development and pathology, which would further advance the pharmacology application of FMOD.

## Author Contributions

CL conceived the opinion. CL, PH, and ZZ wrote and revised the manuscript. WJ and CH edited and proofread the manuscript. CL, ZZ, and MZ supervised the writing process and approved the manuscript. All authors reviewed the final manuscript.

## Conflict of Interest

ZZ is an inventor on fibromodulin-related patents assigned to UCLA. ZZ is a founder of Scarless Laboratories Inc., which sublicenses fibromodulin-related patents from the UC Regents, who also hold equity in the company. ZZ is also a former officer of Scarless Laboratories Inc.

The handling editor is currently organizing a Research Topic with one of the authors ZZ and confirms the absence of any other collaboration.

The remaining authors declare that the research was conducted in the absence of any commercial or financial relationships that could be construed as a potential conflict of interest.
